# Equations for estimating binary mixture toxicity: 3-methyl-2-butanone with a series of electrophiles

**DOI:** 10.1371/journal.pone.0306382

**Published:** 2024-07-03

**Authors:** Douglas A. Dawson, Terry W. Schultz

**Affiliations:** 1 Department of Biological Sciences and Toxicology, Ashland University, Ashland, Ohio, United States of America; 2 College of Veterinary Medicine, University of Tennessee, Knoxville, Tennessee, United States of America; University of East Anglia, UNITED KINGDOM

## Abstract

Mixture toxicity was determined for 32 binary combinations. One chemical was the non-reactive, non-polar narcotic 3-methyl-2-butanone (always chemical A) and the other was a potentially reactive electrophile (chemical B). Bioluminescence inhibition in *Allovibrio fischeri* was measured at 15-, 30-, and 45-minutes of exposure for A, B, and the mixture (MX). Concentration-response curves (CRCs) were developed for each chemical and used to develop predicted CRCs for the concentration addition (CA) and independent action (IA) mixture toxicity models. Also, MX CRCs were generated and compared with model predictions using the 45-minute data. Classification of observed mixture toxicity used three specific criteria: 1) predicted IA EC_50_ vs. CA EC_50_ values at 45-minutes, 2) consistency of 45-minute MX CRC fit to IA, CA, or otherwise at three effect levels (EC_25_, EC_50_ and EC_75_), and 3) the known/suspected mechanism of toxicity for chemical B. Mixture toxicity was then classified into one of seven groupings. As a result of the predicted IA EC_50_ being more toxic than the predicted CA EC_50_, IA represented the greater toxic hazard. For this reason, non-sham MXs having toxicity consistent with CA were classified as being “coincident” with CA rather than mechanistically-consistent with CA. Multiple linear regression analyses were performed to develop equations that can be used to estimate the toxicity of other 3M2B-containing binary mixtures. These equations were developed from the data for both IA and CA, at each exposure duration and effect level. Each equation had a coefficient of determination (r^2^) above 0.950 and a variance inflation factor <1.2. This approach can potentially reduce the need for mixture testing and is amenable to other model systems and to assays that evaluate toxicity at low effect levels.

## Introduction

Chemical mixture toxicity is an active area of environmentally-relevant research [1]. Studies have evaluated environmental contamination [2], organic chemicals [3], heavy metals [4], pharmaceutical presence [5], and habitat impacts [6]. Such studies may focus on a few specific chemicals relevant to situations of concern or include many chemicals of interest.

When many chemicals are present in a mixture at low concentrations relative to their acute aquatic toxicities, their combined effect closely follows concentration addition or simple additivity [7]. This additivity holds even when the chemicals are structurally dissimilar or exhibit different modes of action. However, as the number of chemicals decreases toward binary mixtures, increased variance in additivity is reported [7]. Specifically, binary and complex mixtures of reversible membrane-perturbating chemicals (alkanes, halogenated aliphatics, alcohols, ketones, etc.) tested in bacterial assays indicated additivity in their joint action [8]. In contrast, the toxicity of binary mixtures of non-polar narcotics and reactive aldehydes yielded additive to greater than additive effects in the Microtox assay [9]. Another Microtox study evaluating binary mixtures of reactive toxicants, reported greater than additive effects 18% of the time among chemicals with different mechanisms of toxicity [10]. The authors noted that the slope of a chemical’s concentration-response curve is vital in determining the mode of joint toxic actions.

When assessing the hazard posed by chemical mixtures, several mixture toxicity models are available to provide context for the experimentally-exhibited toxicity. Two commonly used models [11] are concentration addition (CA–a.k.a. dose addition) and independent action (IA–a.k.a. independence). The former model, CA [12], describes the combined effect obtained when the chemicals in the mixture act alike, just as if their molecules were the same substance (as in a sham combination). Hence, CA suggests that the chemicals work at the same molecular site of action; however, it is not definitive but depends on the slopes of the concentration-response curves (CRCs) [13]. Various mathematical approaches, generally derivatives of the Hill equation [14, 15], can be used for calculating CA, each appearing effective for given purposes [16–18]. The IA model [19] is used to describe the toxicity associated with chemicals that act at different molecular sites, thereby resulting in an “unaffected” action when applied with another chemical [13]. Independent action has been suggested to be the appropriate model for quantitatively evaluating potentiation and antagonism [13]. For any given mixture, the resulting combined effect may not be consistent with these models.

The models CA and IA were chosen to evaluate the toxicity of electrophiles in binary combinations [20]. Electrophiles are electron-deficient chemicals that can react with electron-rich chemicals called nucleophiles. As Schwöbel and colleagues [21] summarized, exogenous electrophilic substances are in extremes, either hard with low polarizability or soft with high polarization. When introduced into an organism, electrophiles generally follow the rule of like-reacts-with-like (i.e., soft-with-soft and hard-with-hard). However, many electrophiles are not specific regarding their molecular targets ‐ they can react with different biological nucleophilic targets (e.g., S-, N-, and O-containing moieties). Many nucleophilic target sites are found in biological molecules (e.g., proteins, lipids, DNA). Since the principle of like-reacts-with-like applies, hard biological nucleophiles include DNA and amino groups such as lysine. In contrast, soft nucleophiles include thiol groups such as cysteine. These electro(nucleo)philic interactions, via different mechanisms, result in elevated acute toxicity and cytotoxicity.

There are more than 50 specific mechanisms of reactive biomolecular binding [21, 22]. The particular mechanisms are traditionally grouped into “chemical modes of action” (MOA) [23], such as Michael addition, aromatic nucleophilic substitution (SNAr), bi-molecular nucleophilic substitution (SN2), and Schiff base formation. These MOA describe direct-acting reactions that covalently modify bio-nucleophiles, subsequently leading to apical toxic events [24] and allow for classifying electrophiles into appropriate mechanistic applicability domains associated with particular chemical spaces [22, 23].

This study tested 3-methyl-2-butanone (3M2B) in binary combination with a series of direct-acting electrophiles. In acute aquatic toxicity profiles, 3M2B is consistently reported as a “neutral organic,” “base surface narcotic,” and “class 1 narcotic” [22]. In the model organism *Allovibrio fischeri*, toxicity manifested as bioluminescence inhibition and was determined for each single chemical and mixture at 15-, 30-, and 45-min of exposure. The results of mixture tests were then compared with effects predicted by the CA and IA models. Additionally, multiple linear regression equations for estimating mixture toxicity were developed to reduce the need for mixture testing.

## Materials and methods

### Chemicals, reagents and toxicity testing

Chemicals tested in this study, including abbreviations, Chemical Abstract Service Registry numbers, SMILES structures, log Kow, vapor pressure and chemical reaction mechanisms are presented (Table 1.) Test chemicals were purchased from Aldrich (Milwaukee, WI) or Sigma (St. Louis, MO) in high purity (≥95%) and used without further purification. Dimethyl sulfoxide (DMSO) was used as a carrier solvent; its concentration in test vials was ≤0.1%.

**Table 1:** B-agents for Microtox mixture toxicity studies with 3-methyl-2-butanone.

Rank^a^	Chemical name	Abbr.	CAS#	SMILES^b^	LogKow^c^	Vapor Pressure^d^ (mm Hg 25 C)	Chemical Mechanism of Reactivity^e^^-^^g^
1	1-Bromo-2,4-dinitrobenzene	BDNB	584-48-5	C1 = CC (= C(C = C1[N+] (= O)[O-])[N+] (= O)[O-])Br	2.53	0.0000706	Aromatic nucleophilic substitution (SNAr); two activating groups (NO_2_ or in-ring N) in ortho- or para-positions to the electronegative leaving group (F >Cl >Br >I); **a Br ortho and para to the two NO**_**2**_ **groups**. A proven soft electrophile, experimentally highly reactive with glutathione.; binding potency with cysteine is positive- above 21%, binding potency with lysine is negative- below 9%.
2	1-Chloro-2,4-dinitrobenzene	CDNB	97-00-7	C1 = CC (= C(C = C1[N+] (= O)[O-])[N+] (= O)[O-])Cl	2.17	0.0000849	Aromatic nucleophilic substitution (SNAr); two activating groups (NO_2_ or in-ring N) in ortho- or para-positions to the electronegative leaving group (F >Cl >Br >I); **a Cl ortho and para to the two NO**_**2**_ **groups**. A proven soft electrophile, experimentally highly reactive with glutathione. Experimentally strongly positive in the direct peptide reactivity assay (DPRA), predicted binding potency with cysteine and lysine is above 21%.
3	2,6-Dichloro-4-nitropyridine	26D4NP	25194-01-8	[O-][N+] (= O)C1 = CC(Cl) = NC(Cl) = C1	1.91	0.00221	Aromatic nucleophilic substitution (SNAr); two activating groups (NO_2_ or in-ring N) in ortho- or para-positions to the leaving group; **two Cl both ortho to the in-ring N-atom**. A likely soft electrophile, experimentally highly reactive with glutathione but is not predicted to be reactive with cystine or lysine.
4	2-Chloro-4-nitropyridine	2C4NP	23056-36-2	[O-][N+] (= O)C1 = CC(Cl) = NC = C1	1.27	0.0153	Aromatic nucleophilic substitution (SNAr); two activating groups (NO_2_ or in-ring N) in ortho- or para-positions to the leaving group; **a Cl ortho to the in-ring N-atom**. A probable soft electrophile, unclassified for glutathione reactivity (i.e., basic pH-dependent reactivity) and not predicted to be reactive with cystine or lysine.
5	Ethyl bromoacetate	EBAC	105-36-2	CCOC (= O)CBr	1.12	224	Bimolecular nucleophilic substitution (SN2) at the sp3 carbon atom; leaving group F <<Cl <<Br ≈ I; activating group (-C (= O)-) > (-C (= O)O-) > (-C (= O)NH_2_) ≈ (-C≡N); **a Br beta to the carbonyl group**. A proven soft electrophile, experimentally extremely reactive with glutathione; predicted binding potency with cysteine and lysine is positive- above 21%.
6	3-Chloro-2,4-pentanedione; 3-chloropentane-2,4-dione	3C24P	1694-29-7	CC (= O)C(C (= O)C)Cl	0.23	1.30	Bimolecular nucleophilic substitution (SN2) at the sp3 carbon atom; leaving group F <<Cl <<Br ≈ I; activating group (-C (= O)-) > (-C (= O)O-) > (-C (= O)NH_2_) ≈ (-C≡N); **a Cl beta to both carbonyl groups**. A likely soft electrophile, experimentally highly reactive with glutathione. The structure is out of the domain for cysteine or lysine binding predictions.
7	4-Nitrobenzyl bromide; 1-(bromomethyl)-4-nitrobenzene	4NBB	100-11-8	C1 = CC (= CC = C1CBr)[N+] (= O)[O-]	2.70	0.000977	Bimolecular nucleophilic substitution (SN2) on benzylic carbon atom; leaving group F <<Cl <<Br ≈ I; activating group nitrobenzene; **a Br para to a NO**_**2**_**-benzyl group**. A likely soft electrophile, experimentally highly reactive with glutathione; predicted binding potency with cysteine is positive- above 21%, predicted binding potency with lysine is negative- below 9%.
8	Methyl-2-bromopropionate	M2BP	5445-17-0	CC(C (= O)OC)Br	1.13	6.26	Bimolecular nucleophilic substitution (SN2) at the sp3 carbon atom; F <<Cl <<Br ≈ I; activating group (-C (= O)-) > (-C (= O)O-) > (-C (= O)NH_2_) ≈ (-C≡N); **a Br beta to the carbonyl group**. A likely soft electrophile, experimentally highly reactive with glutathione; predicted binding potency with cysteine and lysine is negative- below 9%.
9	Ethyl propiolate; ethyl prop-2-ynoate	EP	623-47-2	CCOC (= O)C#C	0.58	15.6	Micheal addition (MA) for the activating moiety (R-C (= O)-R), alkynes (C#C) > corresponding alkenes (C = C); terminal acetylenic > internal ethynylene; **an acetylenic group beta to a carbonyl group**. A likely soft electrophile, experimentally highly reactive with glutathione. The structure is out of the domain for cysteine or lysine binding predictions.
10	Chloroacetonitrile; 2-chloroacetonitrile	CLAN	107-14-2	C(C#N)Cl	0.45	0.150	Bimolecular nucleophilic substitution (SN2) at the sp3-Carbon atom; F <<Cl <<Br ≈ I; activating group (-C (= O)-) > (-C (= O)O-) > (-C (= O)NH2) ≈ (-C≡N); **a Cl beta to a nitrile group**. A likely soft electrophile, experimentally moderately reactive with glutathione. The structure is out of the domain for cysteine or lysine binding predictions.
11	3-Methyl-2-butanone; 3-methylbutan-2-one	3M2B	563-80-4	CC(C)C (= O)C	0.84	52.2	Nonpolar narcosis (NPN); baseline toxicity; a proven non-electrophile, experimentally nonreactive with glutathione reactivity at saturation 250mM, predicted cystine and lysine binding is negative- below 9%. The impaired membrane-related processes are due to the nonspecific intercalation of chemicals in biological membranes.
12	Trichloroacetonitrile; 2,2,2-trichloroacetonitrile	TCLAN	545-06-2	C(#N)C(Cl)(Cl)Cl	2.09	74.1	Halogenated nitrile; Bimolecular nucleophilic substitution (SN2) at the sp3 carbon atom; F <<Cl <<Br ≈ I; reactivity increase with number of halogens; activating group (-C (= O)-) > (-C (= O)O-) > (-C (= O)NH2) ≈ (-C≡N); **three Cl groups beta to a nitrile group**. A probable soft electrophile, experimentally not reactive with glutathione at saturation; predicted cysteine binding is positive- above 21%, predicted lysine binding is positive- above 21%.
13	Ethyl acrylate; ethyl prop-2-enoate	EA	140-88-5	CCOC (= O)C = C	1.32	38.6	Beta-unsaturated ester; Micheal addition (MA); for the activating moiety, (R-C (= O)-R), alkynes (C#C) > corresponding alkenes (C = C); terminal vinyl > internal vinylene; unsubstituted alkenes > alkyl-substituted alkenes with α-C monoalkyl-substituted alkenes > β-C monoalkyl-substituted alkenes > α,β-C dialkyl-substituted alkenes; **a vinyl group beta to a carbonyl group**. A proven soft electrophile, highly reactive with glutathione; predicted cystine binding is positive- above 21%, predicted lysine binding is positive—above 21%.
14	Methyl vinyl ketone; but-3-en-2-one	MVK	78-94-4	CC (= O)C = C	0.41	91.3	Beta-unsaturated ketone; Micheal addition (MA); for the activating moiety, (R-C (= O)-R), alkynes (C#C) > corresponding alkenes (C = C); terminal vinyl > internal vinylene; unsubstituted alkenes > alkyl-substituted alkenes with α-C monoalkyl-substituted alkenes > β-C monoalkyl-substituted alkenes > α,β-C dialkyl-substituted alkenes; **a vinyl group beta to a carbonyl group**. A proven soft electrophile, experimentally extremely reactive with glutathione; predicted cystine binding is positive- above 21%, predicted lysine binding is uncertain- between 9% and 21%.
15	2,3-Pentandione; pentane-2,3-dione	23P	600-14-6	CCC (= O)C (= O)C	-0.85	31.1	Alpha-beta-diketone, **α,β-Dicarbonyl** Schiff base former (Di-Sbf); a probable soft electrophile, unclassified for glutathione reactivity (i.e., non-covalent reaction); predicted cysteine binding is positive—above 21%, and predicted lysine binding is positive—above 21%.
16	Ethyl chloroacetate	ECAC	105-39-5	CCOC (= O)CCl	0.94	4.87	Halogenated ester; Bimolecular nucleophilic substitution (SN2) at the sp3 carbon atom; F <<Cl <<Br ≈ I; reactivity increase with number of halogens; activating group (-C (= O)-) > (-C (= O)O-) > (-C (= O)NH2) ≈ (-C≡N); **a Cl beta to a carbonyl group**. A proven soft electrophile, experimentally moderately reactive with glutathione; predicted cysteine binding is positive- above 21%, predicted lysine binding is positive- above 21%.
17	2,3-Butandione; butane-2,3-dione	23B	431-03-8	CC (= O)C(C) = O	-1.34	56.8	Alpha-beta-diketone; **α,β-Dicarbonyl** Schiff base former (Di-Sbf); a probable soft electrophile, unclassified for glutathione reactivity (i.e., non-covalent reaction). Experimentally strongly positive in the direct peptide reactivity assay (DPRA), predicted binding potency with cysteine and lysine is above 21%.
18	Ethyl vinyl ketone; pent-1-en-3-one	EVK	1629-58-9	CCC (= O)C = C	0.90	38.2	Beta-unsaturated ketone; Micheal addition (MA); for the activating moiety, (R-C (= O)-R), alkynes (C#C) > corresponding alkenes (C = C); terminal vinyl > internal vinylene; unsubstituted alkenes > alkyl-substituted alkenes with α-C monoalkyl-substituted alkenes > β-C monoalkyl-substituted alkenes > α,β-C dialkyl-substituted alkenes; **a vinyl group beta to a carbonyl group**. A proven soft electrophile, experimentally extremely reactive with glutathione; predicted cysteine binding is positive- above 21%, predicted lysine binding is uncertain- between 9% and 21%.
19	3-Chloro-2-butanone; 3-chlorobutan-2-one	3C2B	4091-39-8	CC(C (= O)C)Cl	0.44	19.5	Halogenated ketone; Bimolecular nucleophilic substitution (SN2) at the sp3 carbon atom; F <<Cl <<Br ≈ I; activating group (-C (= O)-) > (-C (= O)O-) > (-C (= O)NH2) ≈ (-C≡N); **a Cl beta to the carbonyl group**. A likely soft electrophile, experimentally moderately reactive with glutathione. The structure is out of the domain of cysteine or lysine predictions.
20	Methyl crotonate; methyl (E)-but-2-enoate	MC	623-43-8	C/C = C/C (= O)OC	1.14	17.1	Beta-unsaturated ester; Micheal addition (MA); for the activating moiety, (R-C (= O)-R), alkynes (C#C) > corresponding alkenes (C = C); terminal vinyl > internal vinylene; unsubstituted alkenes > alkyl-substituted alkenes with α-C monoalkyl-substituted alkenes > β-C monoalkyl-substituted alkenes > α,β-C dialkyl-substituted alkenes; **a vinylene group beta to a carbonyl group**. A likely soft electrophile, experimentally moderately reactive with glutathione. The structure is out of the domain of cysteine or lysine predictions.
21	3,4-Hexandione; hexane-3,4-dione	34H	4437-51-8	CCC (= O)C (= O)CC	-0.35	12.3	Alpha-beta-diketone; **α,β-Dicarbonyl** Schiff base former (Di-Sbf); a probable soft electrophile that is unclassified for glutathione reactivity (i.e., non-covalent reaction); predicted cysteine binding is positive- above 21%, predicted lysine binding is positive- above 21%.
22	Methyl-2-chloroacetoacetate; methyl 2-chloro-3-oxobutanoate	M2CA	4755-81-1	CC (= O)C(C (= O)OC)Cl	-0.51	0.956	Halogenated ester; Bimolecular nucleophilic substitution (SN2) at the sp3 carbon atom; F <<Cl <<Br ≈ I; activating group (-C (= O)-) > (-C (= O)O-) > (-C (= O)NH2) ≈ (-C≡N); **a Cl beta to a carbonyl group**. A likely soft electrophile, experimentally highly reactive with glutathione; predicted cysteine and lysine binding are negative ‐ below 9%.
23	Diethyl maleate; diethyl (Z)-but-2-enedioate	DEM	141-05-9	CCOC (= O)/C = C\C (= O)OCC	2.20	0.178	Beta-unsaturated ketone; Micheal addition (MA); for the activating moiety, (R-C (= O)-R), alkynes (C#C) > corresponding alkenes (C = C); terminal vinyl > internal vinylene; unsubstituted alkenes > alkyl-substituted alkenes with α-C monoalkyl-substituted alkenes > β-C monoalkyl-substituted alkenes > α,β-C dialkyl-substituted alkenes; trans isomer > cis isomer; **a vinylene group beta to two carbonyl groups**. A proven soft electrophile, experimentally extremely reactive with glutathione; predicted cysteine binding is positive- above 21%, predicted lysine binding is positive- above 21%.
24	Bromoacetonitrile; 2-bromoacetonitrile	BRAN	590-17-0	C(C#N)Br	0.20	4.33	Halogenated nitrile; Bimolecular nucleophilic substitution (SN2) at the sp3 carbon atom; F <<Cl <<Br ≈ I; activating group (-C (= O)-) > (-C (= O)O-) > (-C (= O)NH2) ≈ (-C≡N); **a Br beta to a nitrile group**. A likely soft electrophile, experimentally highly reactive with glutathione. The structure is out of the domain of cysteine or lysine predictions.
25	Ethyl fluoroacetate	EFAC	459-72-3	CCOC (= O)CF	0.80	15.6	Halogenated ester; **No alerts found**; suspected bimolecular nucleophilic substitution (SN2) at the sp3 carbon atom; F <<Cl <<Br ≈ I; activating group (-C (= O)-) > (-C (= O)O-) > (-C (= O)NH2) ≈ (-C≡N); **a F beta to a carbonyl group**. A proven non-electrophile, nonreactive with glutathione reactivity at 250mM, predicted cystine and lysine binding is negative- below 9%.
26	Iodoacetonitrile; 2-iodoacetonitrile	IAN	624-75-9	C(C#N)I	0.61	0.757	Halogenated nitrile; Bimolecular nucleophilic substitution (SN2) at the sp3 carbon atom; F <<Cl <<Br ≈ I; activating group (-C (= O)-) > (-C (= O)O-) > (-C (= O)NH2) ≈ (-C≡N); **a I beta to a nitrile group**. A likely soft electrophile, experimentally highly reactive with glutathione. The structure is out of the domain of cysteine or lysine predictions.
27	2-Hydroxyethylacrylate; 2-hydroxyethyl prop-2-enoate	2HEA	818-61-1	C = CC (= O)OCCO	-0.21	0.0523	Beta-unsaturated ester; Micheal addition (MA); for the activating moiety, (R-C (= O)-R), alkynes (C#C) > corresponding alkenes (C = C); terminal vinyl > internal vinylene; unsubstituted alkenes > alkyl-substituted alkenes with α-C monoalkyl-substituted alkenes > β-C monoalkyl-substituted alkenes > α,β-C dialkyl-substituted alkenes; **a vinyl group beta to a carbonyl group**. A proven soft electrophile, experimentally highly reactive with glutathione. Experimentally strongly positive in the direct peptide reactivity assay (DPRA), predicted binding potency with cysteine and lysine is above 21%.
28	Hydroxypropyl methacrylate; 2-hydroxypropyl 2-methylprop-2-enoate	HPM	27813-02-1	CC(COC (= O)C (= C)C)O	0.95	0.0724	Beta-unsaturated ester; Micheal addition (MA); for the activating moiety, (R-C (= O)-R), alkynes (C#C) > corresponding alkenes (C = C); terminal vinyl > internal vinylene; unsubstituted alkenes > alkyl-substituted alkenes with α-C monoalkyl-substituted alkenes > β-C monoalkyl-substituted alkenes > α,β-C dialkyl-substituted alkenes; **an electronically hindered vinyl group beta to a carbonyl group**. A likely soft electrophile, experimentally slightly reactive with glutathione. Experimentally weakly positive in the direct peptide reactivity assay (DPRA), predicted cysteine binding is positive- above 21%, predicted lysine binding is negative- below 9%.
29	Diethyl sulfate	DES	64-67-5	CCOS (= O) (= O)OCC	1.14	0.212	Alkyl sulfate; bimolecular nucleophilic substitution (SN2) substitution at the sp3 carbon; **an OS (= O) (= O)OR moiety as the leaving group**. A likely soft electrophile, experimentally moderately reactive with glutathione; predicted cysteine binding is positive- above 21%; predicted lysine binding is uncertain- between 9 and 21%.
30	Dimethyl sulfate	DMS	77-78-1	COS (= O) (= O)OC	0.16	0.667	Alkyl sulfate; Bimolecular nucleophilic substitution (SN2) substitution at the sp3 carbon; **an OS (= O) (= O)OR moiety as the leaving group**. A likely soft electrophile, experimentally moderately reactive with glutathione; predicted cysteine binding is positive- above 21%; predicted lysine binding is uncertain- between 9% and 21%.
31	Butyl glycidyl ether; 2-(butoxymethyl)oxirane	BGE	2426-08-6	CCCCOCC1CO1	0.63	3.20	Cyclic ether; Epoxide ring-opening bi-molecular nucleophilic substitution reaction (ERO-SN2); A probable soft electrophile, experimentally slightly reactive with glutathione; predicted cysteine binding is positive- above 21%, predicted lysine binding is uncertain- between 9% and 21%.
32	4-Vinylpyridine; 4-ethenylpyridine	4VP	100-43-6	C = CC1 = CC = NC = C1	1.71	1.70	Pyridine; Michael addition (MA); aromatic activated double bond. Profiling in the OECD QSAR Toolbox V 4.5 gives mixed results. In early aquatic toxicity classification schemes, 4VP is classified as Class 1 narcotic, baseline toxicant, or neutral organic; Subsequently, it was classified as reactive unspecified or non-categorized. 4VP is Ames mutagenicity positive, CHO clastogenicity positive, and a skin sensitizer. A proven soft electrophile, experimentally highly reactive with glutathione; predicted cysteine binding is positive- above 21%, predicted lysine binding is negative- below 9%.

^a^ Rank is listed from most to least toxic as predicted by the 45-min EC_50_ for independent action (IA).

^b^ SMILES, Simplified Molecular-Input Line-Entry System notation of chemical structure.

^c^ Log Kow (1-octanol/waterer partition coefficient) values were secured from EPI Suite v4.11.

^d^ Vapor pressure values were secured from EPI Suite v4.11.

^e^ Chemical mechanism of reactivity was derived following the descriptions within [23–25].

^f^ in chemico glutathione reactivity was assessed following the protocol in [26]; experimental values were reported in [22].

^g^ Direct peptide reactivity assay predictions followed the protocol in [27]; experimental values were reported by [28], and predictions were made from [22].

Freeze-dried bacterial reagent, Microtox diluent, and the bacterial reconstitution solution were obtained from Modern Water (New Castle, DE). Vials of bacterial reagent were kept frozen at −20°C before a 20-minute reconstitution period just prior to test initiation. For each given combination, separate bacterial reagent vials were used to test each chemical alone and the mixture.

The marine bacterium *Allovibrio fischeri* was the model organism, with bioluminescence inhibition being measured with a Microtox analyzer. The acute toxicity testing procedures were noted previously [30]. For each binary combination, each chemical was tested alone, denoted as chemical A (always 3M2B) or B (an electrophile), and the A+B mixture (MX). Each test had seven duplicated concentrations and a duplicated control. Test concentrations were prepared via serial dilution in mg/L and later converted to μM. Depending primarily on B’s toxicity change over time, one of three dilution factors (1.75, 1.867, or 2.0) was used in testing. The dilution factor was kept the same for all tests of a given combination.

Initial light readings for each control and treatment vial were taken before chemical exposure. Toxicity assessments were made after 15-, 30-, and 45-minutes of exposure. During testing, treatment vials were held at 15°C ± 0.2°C.

### Procedures for curve-fitting and other calculations

Microtox software collected data and converted light readings to percent effect values. The data were input into SigmaPlot (v. 15.0; Inpixon, Palo Alto, CA) and evaluated via user-developed program files. Raw data were fitted to sigmoid curves with a five-parameter logistic function from which the minimum effect parameter had been removed [17]. The remaining parameters were maximum effect, EC_50_, slope, and asymmetry (s). This modified function was designated 5PL-1P to delineate it from the software’s standard four and five-parameter logistic functions.

Curve fitting was performed using Eq (1):

$$y=max/1+{\left(\frac{xb}{x}\right)}^{Hillslope^{s}}$$ (1)

in which y = % effect, max = maximum effect, x = concentration, and s = asymmetry. The variable xb was determined using Eq (2).


$$xb=EC50\times 10^{\frac{1}{\;Hillslope}}\;*\;log2^{1/s}-1$$ (2)

Initial parameters for these regressions were estimated automatically. The following constraints were used for data fitting: a) EC_50_ > 0, b) 0.1 < s < 10, c) max = 100.

For all concentration–response data, the following effective concentration values: EC_25_, EC_50_, and EC_75_, as well as slope, asymmetry (s), maximum effect, and coefficient of determination (CD or r^2^) values were calculated for A, B, and MX at each exposure duration. For the B and MX tests, chemical concentrations were converted to 3M2B-equivalents via the B factor from Eq (3) [29]:

$$\left[B_{f}\right]=[A]/[B].$$ (3)

Time-dependent toxicity (TDT) values were calculated by Eq 4:

TDT=((15minEC50−45minEC50)/(15minEC50×0.667))×100 (4)

to give a percentage-based value [30]. These calculations were made separately for each combination of A, B, and MX.

Calculation procedures for obtaining predicted CRCs for the CA and IA models have been described [31]. When A and B are equally effective in CA, the CA EC_50_ is left-shifted (when viewed graphically) by a dose-ratio (DR) factor of two. The CA_50_ and DR were calculated using Eqs 5 and 6, respectively.


CA50=a50/DR50 (5)

Herein, CA_50_ is the EC_50_ for CA, a_50_ is the EC_50_ of the more potent single chemical, and b_50_ is the EC_50_ of the less potent single chemical.


$$DR50=1+\left(\frac{a50}{b50}\right)$$ (6)

Therefore, when a_50_ = b_50_ the DR_50_ = 1 + (1) = 2, that is, the CA_50_ = a50/2. This approach allows one to calculate the predicted CA EC_50_ when A and B are not exactly equally effective (very common) using the calculated DR to adjust the predicted CA value. For example, suppose A has an EC_50_ of 52.4 μM and B has an A-equivalent concentration EC_50_ of 61.7 μM. The DR is 1 + (52.4/61.7) = 1.8493. In this example, A was the more potent agent, so the EC_50_ for A was divided by this DR value to give the CA EC_50_ of 28.3 μM. Calculations of the EC_25_ and EC_75_ values for the predicted CA curve were performed in this same manner. This approach allows the DR to be adjusted at different EC_x_ levels (i.e., EC_25_, EC_50_, and EC_75_) and, for example, in situations in which A is more potent than B at the EC_25_, but B is more potent than A at the EC_50_ and EC_75_. Taken together, the predicted CA values for EC_25_, EC_50_, and EC_75_, as well as the CA maximum effect value (Eq 7) allow calculating the predicted CA curve using the 5PL-1P curve fitting procedure noted above.


max=a50×100x (7)

Theoretical curves for the IA model were developed using Eq 8:

$$yA+\left(yB\times \frac{100-yA}{100}\right)$$ (8)

with yA and yB being percent effect values for A and B, respectively.

For each combination and exposure duration, the three EC_x_ values were calculated for A, B, MX, and for the predicted CA and IA curves. Concentration addition quotient (AQ) values were calculated via Eq 9:

AQx=MXECx/CAECx (9)

in which the subscript x can represent either the 25, 50 or 75% effect levels. Likewise, independent action quotient (IQ) values were calculated using Eq 10:

IQx=MXECx/IAECx (10)

Final mixture toxicity determinations vs. the CA and IA models were made for each combination by determining: 1) whether the predicted 45-min IA EC_50_ was more toxic than that for CA, 2) whether the three 45-min AQ_x_ or IQ_x_ values, respectively, were from 0.90 to 1.10, and 3) by giving due consideration to the mechanisms of toxic action for A and B (as per Table 1).

### Statistical analyses

Statistical tests within SigmaPlot were used to analyze the data further. In the study, 32 binary combinations were tested; for each 3M2B served as chemical A. Since one combination was a sham (i.e., 3M2B with 3M2B), there were a total of 33 tests of 3M2B alone. To determine the repeatability of the 3M2B-alone tests, the mean, standard deviation, coefficient of variation (CV), range, and Shapiro-Wilk W values were calculated for each of the following: EC_25_, EC_50_, EC_75_, slope, asymmetry (s), coefficient of determination (CD or r^2^) and TDT. The Shapiro-Wilk test evaluated the fitting of sample quartiles to standard normal quartiles [32].

Simple linear regression analyses were performed within SigmaPlot to delineate any correlations between the 45-min MX EC_50_ values and the following: 45-min EC_50_ values for A-alone (3M2B), B-alone (both in 3M2B-equivalent concentrations and actual B concentrations ‐ all in μM), AQ_50_, IQ_50_ and the 15- to 45-min TDT values for B-alone.

Testing of 32 binary mixtures containing 3M2B offered an opportunity to develop equations to estimate mixture toxicity for other 3M2B-containing mixtures not tested herein. This was done by multiple linear regression (MLR) within SigmaPlot, using test data for MX EC_x_ as the dependent variable and either the CA EC_x_ and AQ_x_ or the IA EC_x_ and IQ_x_ as independent variables at each exposure duration. For each equation, CD (r^2^), standard error of the estimate (SEE), and variance inflation factor (VIF) values were included. The VIF assessed the likelihood of collinearity between independent variables, with values <5.0 having low concern for collinearity [33].

## Results and discussion

While toxicity was determined for three exposure durations (15-, 30-, and 45-min), for space considerations detailed results are presented primarily for the latter timepoint.

The repeatability of results for Microtox testing was evaluated by examining the results from all 3M2B-alone tests (Table 2). Therein, CV values below 20 were obtained for each mean EC_x_ value and each mean slope, CD, and TDT value. Asymmetry (s) values for 3M2B had more variable means, but CV values remained below 40, thereby being acceptable for test-to-test variation [34]. In addition, the W statistic from the Shapiro-Wilk test denoted the fitting of sample quartiles to standard normal quartiles. Sample values with a W score = 1 represent a perfect fit [32]. For 3M2B, W values ranged from 0.771 to 0.967, with 13 of 19 endpoints above 0.900, including all nine EC_x_ values.

**Table 2:** Statistics for 3-methyl-2-butanone (3M2B) alone (n = 33).

Parameter	Time (min)	Mean	Std. dev.	CV^a^	Range	SW-W^b^
EC_25_^c^	15	140.16	24.1	17.2	85.8	0.925
	30	146.53	27.1	18.5	105.0	0.940
	45	152.38	26.7	17.5	103.2	0.943
EC_50_^c^	15	442.05	62.0	14.0	241.0	0.931
	30	441.38	72.9	16.5	326.8	0.917
	45	461.85	73.4	15.9	319.7	0.944
EC_75_^c^	15	1446.52	189.2	13.1	676.8	0.930
	30	1467.13	219.9	15.0	898.7	0.932
	45	1492.50	240.0	16.1	994.0	0.944
slope	15	0.792	0.066	8.3	0.35	0.800
	30	0.826	0.079	9.6	0.49	0.881
	45	0.883	0.068	7.7	0.39	0.840
s^d^	15	2.34	0.91	39.1	3.67	0.910
	30	1.87	0.73	39.0	3.62	0.895
	45	1.46	0.50	34.2	2.94	0.771
CD^e^	15	0.9985	0.0010	0.10	0.004	0.859
	30	0.9985	0.0009	0.09	0.003	0.904
	45	0.9984	0.0009	0.09	0.004	0.926
TDT^f^	15–45	-14.1	9.63	6.8	44.1	0.967

^a^ CV: coefficient of variation

^b^ SW-W: Shapiro-Wilk W–goodness of fit test

^c^ concentrations are μM 3M2B

^d^ s: asymmetry

^d^ CD–coefficient of determination (r^2^)

^f^ TDT: 15 to 45-min time-dependent toxicity value at EC_50_

For each combination, 45-min EC_50_ values for MX, A, and B (the latter given as both 3M2B-equivalent and actual B concentrations), the calculated 45-min AQ_50_ and IQ_50_, and the 15 to 45-min TDT values are provided (Table 3). For comparative purposes, the MX EC_50_ values are listed within the table from the most toxic to the least toxic combination. Simple linear regressions conducted for the MX EC_50_ values vs. those from each other data column resulted in r^2^ values < 0.700, indicating no strong linear correlations between the MX data and the different variables.

**Table 3:** 45-min toxicity, quotient and agent B TDT values for each combination.

A-B^a^	MX EC_50_^b^	A EC_50_	AQ_50_^c^	B EC_50_	B EC_50_^d^	IQ_50_^c^	B TDT^e^
3M2B-BDNB	105.6	361.4	1.16	121.7	2.0	1.38	114.8
3M2B-26D4NP	115.0	384.0	0.86	203.6	0.3	1.06	54.9
3M2B-EBAC	151.7	488.4	0.87	272.1	1.4	1.08	118.4
3M2B-CDNB	153.9	432.7	1.22	178.1	5.1	1.45	115.2
3M2B-2C4NP	156.4	376.5	1.05	246.4	0.5	1.19	52.5
3M2B-3C24P	158.2	541.5	0.99	227.4	4.9	1.06	94.9
3M2B-ECAC	177.6	497.0	0.77	431.7	303.5	0.99	92.7
3M2B-TCLAN	178.1	565.9	0.95	280.4	6.7	1.10	43.1
3M2B-M2BP	185.9	398.6	1.04	323.1	83.3	1.23	75.8
3M2B-EP	187.4	389.3	1.03	341.8	5.0	1.19	107.9
3M2B-MC	194.2	526.8	0.84	411.7	1770.9	0.93	21.9
3M2B-EA	194.6	399.2	0.96	415.8	894.2	1.17	58.3
3M2B-MVK	204.5	418.7	1.11	327.5	2.7	1.23	99.0
3M2B-4NBB	208.9	467.5	1.07	332.8	0.8	1.39	99.9
3M2B-3M2B	214.5	382.6	1.08	411.3	411.3	1.35	-25.1
3M2B-3C2B	218.2	523.5	0.95	411.4	138.6	1.07	55.1
3M2B-CLAN	233.2	501.4	1.36	261.4	596.4	1.47	106.7
3M2B-2HEA	245.9	498.7	1.00	488.7	604.1	1.05	95.1
3M2B-EVK	247.4	398.1	1.13	483.0	3.3	1.24	94.2
3M2B-23B	260.1	374.0	1.19	526.9	2811.4	1.31	85.4
3M2B-HPM	263.4	505.9	0.82	878.9	2908.2	0.94	-8.0
3M2B-EFAC	264.7	536.3	0.88	684.2	11107.3	1.18	3.7
3M2B-34H	266.3	344.4	1.04	983.0	1978.0	1.25	27.6
3M2B-M2CA	285.7	451.8	1.17	527.6	3.5	1.32	110.6
3M2B-BRAN	286.3	510.9	1.22	434.5	7.3	1.28	104.7
3M2B-IAN	290.4	482.0	1.22	470.4	5.7	1.28	101.0
3M2B-23P	302.9	382.9	1.45	459.7	2636.8	1.71	41.9
3M2B-DEM	311.8	473.2	1.20	576.5	240.3	1.41	51.6
3M2B-DES	332.4	486.9	1.01	1002.2	279.9	1.18	-26.6
3M2B-BGE	371.4	664.1	1.05	751.5	4143.7	1.23	-14.6
3M2B-DMS	390.1	510.9	1.14	1045.3	356.9	1.29	21.7
3M2B-4VP	397.5	550.9	1.19	845.9	57.7	1.28	17.4
r^2^ vs. MX EC_50_^f^		0.171	0.139	0.685	0.016	0.074	0.228

^a^ A:3-methyl-2-butanone (3M2B), B: an electrophile (see Table 1), MX: A+B mixture

^b^ concentrations are given as 3M2B-equivalent values in μM–unless noted otherwise

^c^ values are unitless

^d^ actual B-alone concentrations (μM) before conversion to 3M2B equivalent values

^e^ percent difference in toxicity for actual B-alone concentrations from 15- to 45-min

^f^ coefficient of determination for simple linear regressions

When examining MX toxicity vs. the IA model at 45-min, non-sham MX toxicity was less than that predicted by IA for all combinations except for 3M2B-HPM (Table 4). When IQ_x_ values were tabulated for individual IQ_x_ effect levels, there were four IQ_25_ values <0.90 (lowest = 0.86), twelve were IA consistent, and sixteen were >1.10 (highest = 1.87). For the IQ_50_, nine were IA consistent, the rest were >1.10 (high = 1.71). For the IQ_75_, three were IA consistent, and the rest were >1.10 (highest = 2.14). These data are available at DOI: 10.17605/OSF.IO/2NVDW.

**Table 4:** 45-min non-sham mixture toxicity designations vs. IA and CA models^a^.

Mixture Toxicity vs. IA and CA	Agent B^b^				
Consistent with IA; more toxic than CA	HPM				
Crosses^c^ IA; more toxic than CA	26D4NP				
Crosses IA; crosses CA	EFAC				
Less toxic than IA; more toxic than CA	EBAC	ECAC			
Less toxic than IA; ‘coincident’^d^ with CA	2C4NP 2HEA	3C2B 3C24P	34H DES	EA EP	TCLAN
Less toxic than IA; crosses CA	4NBB	MC			
Less toxic than IA and CA	23B	BDNB	CDNB	DMS	M2BP
	23P	BGE	CLAN	EVK	M2CA
	4VP	BRAN	DEM	IAN	MVK

^a^ IA–Independent action, CA–concentration addition

^b^ mixtures are listed by chemical B abbreviation; chemical A was always 3M2B

^c^ the MX curve had IQ_x_ and/or AQ_x_ values that crossed the IA and/or CA range, respectively

^d^ designation indicates the predicted IA EC_50_ was more toxic than the predicted CA EC_50_

For the CA model, before determining the combined effect of the 45-min MX data, the following points were addressed: 1) was the predicted IA EC_50_ more toxic than the predicted CA EC_50_, 2) were all three AQ_x_ values (x includes 25, 50 or 75% effect levels) from 0.90 and 1.10, and 3) were the ‘B’ chemicals in each combination known or suspected to have the same toxic mechanism as 3M2B.

**Fig 1:**
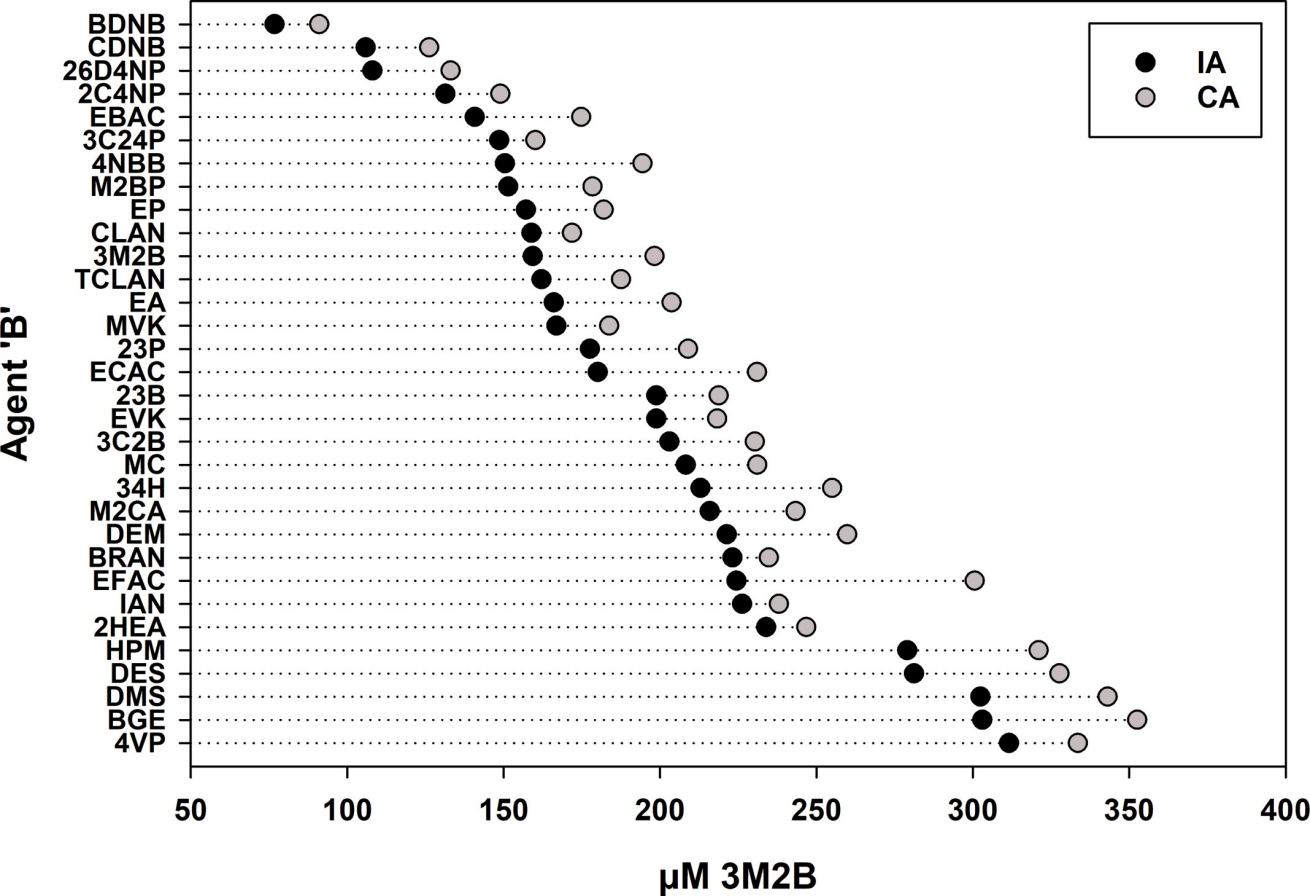
Comparative plot of predicted independent action (IA) and concentration addition (CA) EC_50_ values after 45-min exposures. Predicted IA toxicity was always greater (i.e., at a lower 3M2B-equivalent concentration) than predicted CA toxicity. The combinations are listed on the Y-axis simply as agent ‘B’, since 3M2B was always agent ‘A’. The predicted IA EC_50_ values are shown from most toxic to least toxic.

To address the first point, the toxicities of the predicted 45-min IA EC_50_ and CA EC_50_ values were compared graphically for each combination. This was done by ordering the IA EC_50_ data from most to least toxic on the Y-axis (Fig 1). Therein, it can be seen that the predicted IA EC_50_ was more toxic than the predicted CA EC_50_ for all 32 combinations. Thus, the IA model represents the greater toxic hazard for all MXs in this study. This is likely because 45-min CRC slopes for A and MX were mostly <1.6 [13]. All 32 A-alone tests and 28 of 32 MX tests had slopes <1.6. Of the latter, only two had a slope >1.7. These data are available at DOI: 10.17605/OSF.IO/2NVDW. Comparatively, 17 B-alone CRCs had slopes <1.7, while 15 CRCs had slopes >1.7, with all but two of those being <2.5. Therefore, it was likely that all non-sham mixtures fitting the CA designation at 45-min (see Table 4) had toxicity that was “coincident” with CA [13].

To address the second point, nine non-sham combinations had all three AQ_x_ values from 0.90 to 1.10. Moreover, five other combinations had at least two of the three AQ_x_ values below 0.90 (i.e., a greater-than CA combined effect). However, none of those showed toxicity greater than that predicted by the IA model. This is additional evidence for mixture toxicity being “coincident” with CA.

The third point was addressed by noting that all B agents (except for 3M2B and EFAC) are known/suspected to have a reactive mechanism of toxicity. At the same time, 3M2B is considered a non-reactive, non-polar narcotic (see Table 1). Therefore, mechanistically it is also unlikely that each non-sham mixture having AQ_x_ values fitting the CA designation (i.e., 0.90 ≤ MX EC_x_ ≤ 1.10) were truly CA mechanistically. One should note that all chemicals exhibit a log Kow-determined, reversible, and non-covalent biomembrane fluidity change that alters cellular function (i.e., bioluminescence) [35]. This reversible inhibition may be superseded by covalent reactivity with cellular proteins, especially cystine-rich functional proteins and lysine-rich structural proteins. It is deemed likely that toxic potency for reactive electrophiles may be correlated to reactive mechanism and/or reaction rates.

The results do not provide clear insights into how mixture toxicity is related to the mechanism/mode of toxic action. However, upon examining the listed order of the B-agents in Fig 1 and Table 1 (i.e., from greatest to least toxic as predicted by 45-min IA EC_50_ toxicity) and in Table 3 (from greatest to least observed toxicity at the 45-min MX EC_50_) one can see that the upper and lower chemicals in each listing are similar. As shown in Table 1, SNAr reactive chemicals (i.e., BDNB, CDNB, 26D4NP, and 2C4NP) are among the most toxic when given with 3M2B. Likewise, several chemicals (e.g., HPM, BGE, DES, DMS), that are slightly or weakly reactive with glutathione, are among the least toxic with 3M2B (Tables 1 and 3). The most notable exception is 4VP, a well-studied directing-acting electrophile (see Table 1) that was the least toxic with 3M2B (Table 3).

The toxic effect, inhibition of bioluminescence, and the short duration of the assay advocate that membrane interrelation and covalent binding to soft nucleophiles (i.e., function proteins) are the most likely MOAs. Since 3M2B is non-electrophilic and a classic baseline toxicant, its MOA is exclusively reversible membrane perturbation. The minimal changes in the mean 3M2B potency values with time (see Table 2) indicate that this MOA acts rapidly. Except for EFAC, which was unreactive experimentally, the remaining 30 chemicals demonstrated some degree of soft electrophilicity (see Table 1). However, from Table 1, neither the weight of evidence (i.e., proven, likely, probable, or suspected) nor the potency (i.e., extreme, high, moderate, slight, weak) was related to the rank order for MX toxicity (Table 3).

**Fig 2:**
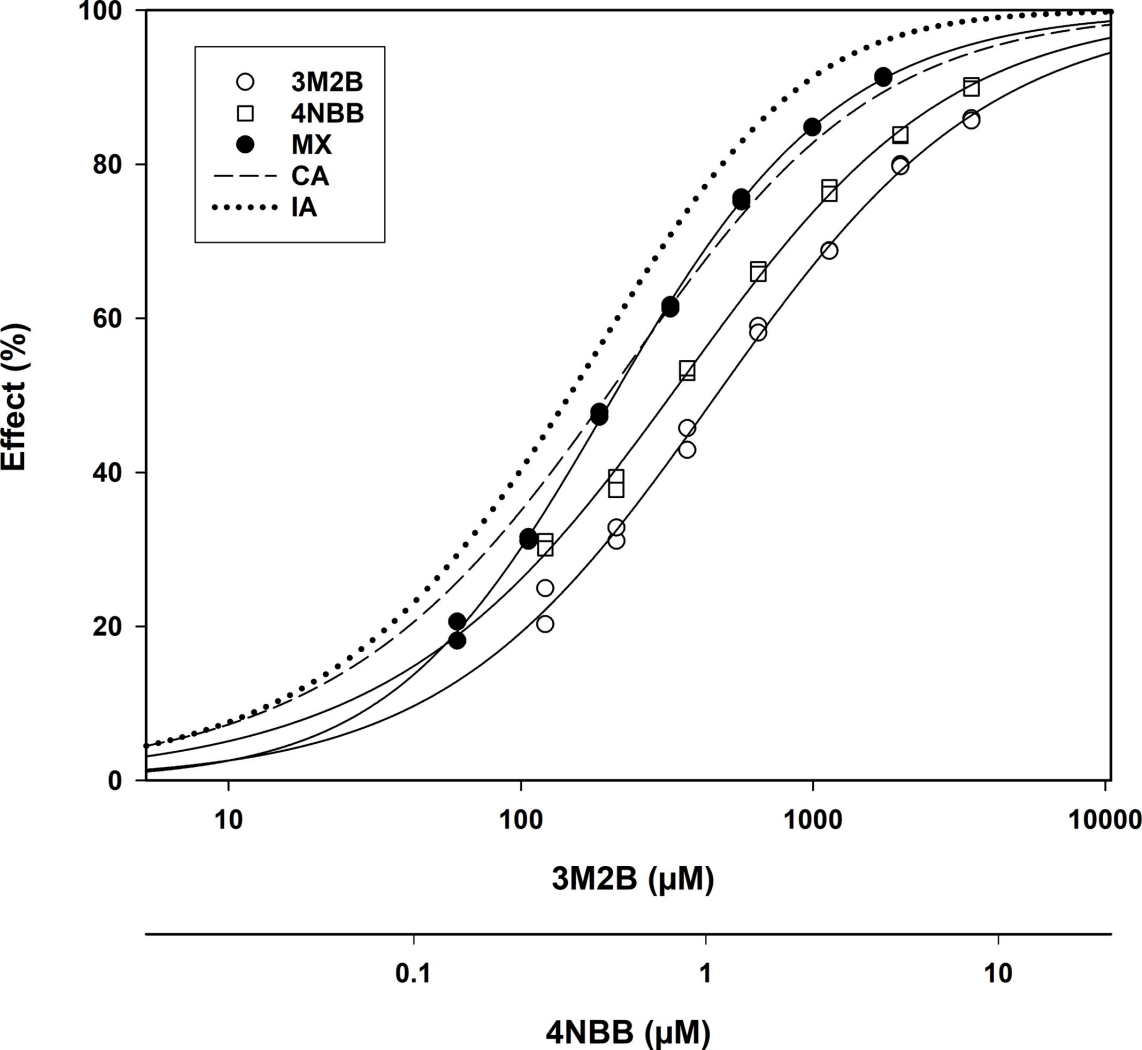
Concentration-response curve plot. The plotted curves are for 3-methyl-2-butanone (3M2B) alone, 4-nitrobenzyl bromide (4NBB) alone, the 3M2B-4NBB mixture and the predicted concentration addition (CA) and independent action (IA) models. Note the crossing of the MX CRC with the predicted CA CRC. All CRCs are given in 3M2B concentrations (the upper X-axis). The lower X-axis depicts the CRC for actual 4NBB alone concentrations.

The 45-min combined effects for each combination as categorized, included instances in which the actual MX CRC crossed over the predicted CA and/or IA CRC. An example of one such instance is presented (Fig 2). This phenomenon suggests a difference in biological action for the chemicals in a given mixture.

**Fig 3:**
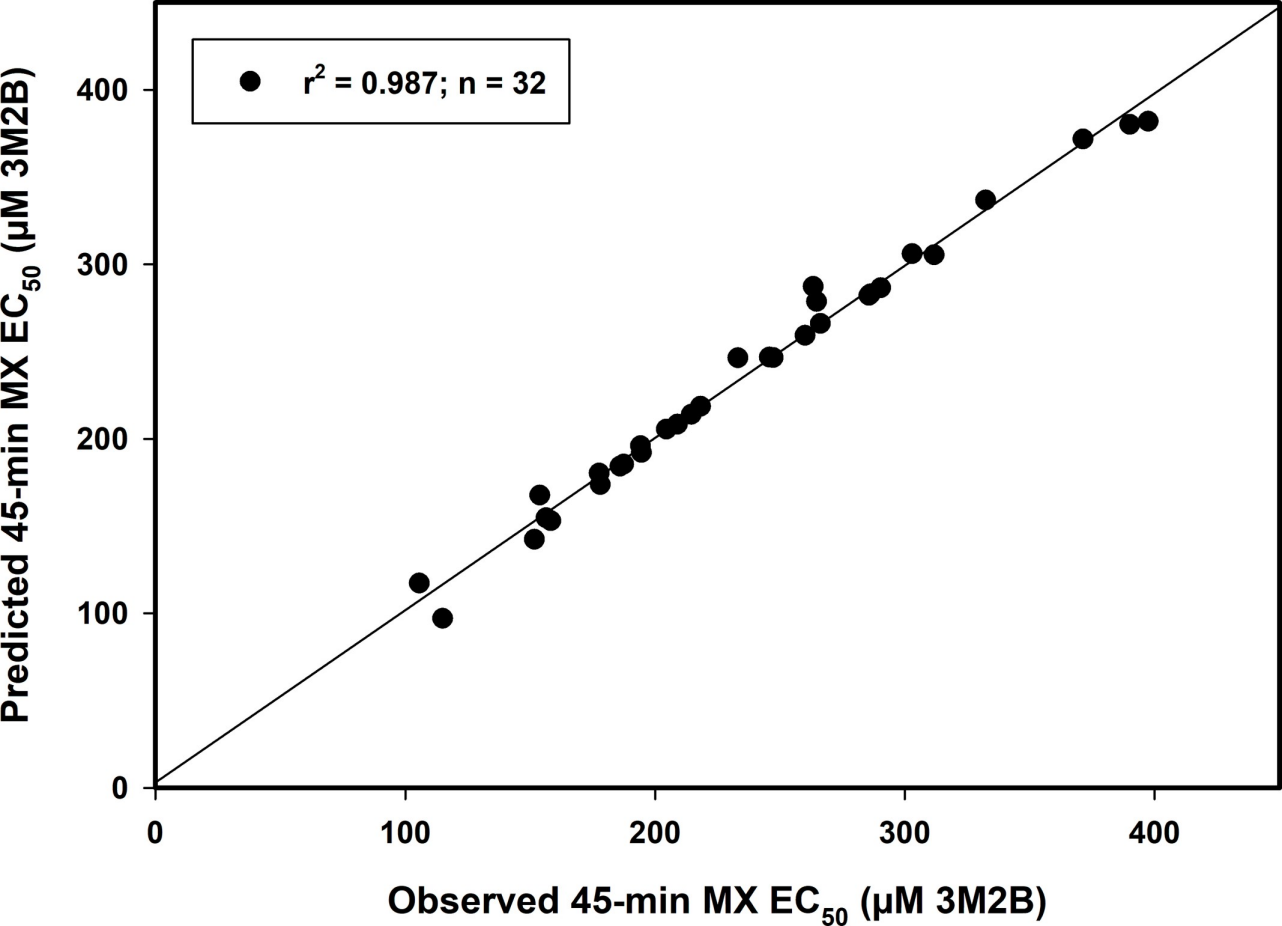
Linear regression plot of observed vs. predicted mixture toxicity for binary combinations containing 3M2B. The 45-min CA EC_50_ and AQ_50_ values generated in this study were inserted into the equation: MX EC_50_ = -233.926 + (1.063 * CA EC_50_) + (219.256 * AQ_50_) (see Table 5) to generate the predicted 45-min MX EC_50_ values for each combination. The predicted MX EC_50_ values were then plotted against the observed 45-min MX EC_50_ values.

Multiple linear regression equations were generated for the IA and CA models at the EC_25_, EC_50_, and EC_75_ at each exposure duration (Table 5 and Fig 3). Each had an r^2^ >0.950 and a VIF <1.2. These results suggest that the approach has utility in estimating mixture toxicity for 3M2B-containing binary combinations that were not tested herein. While these equations only directly apply to the model organism used in this study, conceptually, such MLR equations can be generated for other model organisms, reducing the need for actual mixture testing. Once one has the MLR equation for a given A, the established A-alone data, and the B-alone data for any additional B chemical (preferably with the same dilution factor) can be used to generate predicted CA and IA EC_x_ values. Then, the respective AQ_x_ or IQ_x_ values of interest can be inserted into the appropriate equation to obtain the MX EC_x_ estimate.

**Table 5:** Multiple linear regression equations for estimating mixture toxicity for 3M2B-containing binary mixtures^a^.

Time^b^	IA ‐ Multiple Linear Regression Equations	CD^c^	SEE^d^	VIF^e^
15	MXEC25=−89.319+(1.046×IAEC25)+(84.469×IQ25)	0.986	3.163	1.079
30	MXEC25=−74.708+(1.061×IAEC25)+(69.894×IQ25)	0.994	2.202	1.043
45	MXEC25=−68.819+(1.086×IAEC25)+(63.531×IQ25)	0.990	2.921	1.004
15	MXEC50=−269.008+(1.172×IAEC50)+(227.359×IQ50)	0.988	7.669	1.107
30	MXEC50=−233.279+(1.237×IAEC50)+(186.145×IQ50)	0.988	7.670	1.067
45	MXEC50=−228.200+(1.234×IAEC50)+(183.833×IQ50)	0.989	8.053	1.019
15	MXEC75=−846.300+(1.416×IAEC75)+(590.173×IQ75)	0.986	26.27	1.089
30	MXEC75=−780.760+(1.532×IAEC75)+(504.956×IQ75)	0.970	38.42	1.045
45	MXEC75=−694.802+(1.504×IAEC75)+(456.516×IQ75)	0.958	48.03	1.042
**Time**	**CA ‐ Multiple Linear Regression Equations**	**CD**	**SEE**	**VIF**
15	MXEC25=−99.540+(1.052×CAEC25)+(94.630×AQ25)	0.990	2.685	1.000
30	MXEC25=−83.003+(1.072×CAEC25)+(77.179×AQ25)	0.994	2.082	1.002
45	MXEC25=−75.604+(1.068×CAEC25)+(71.207×AQ25)	0.992	2.661	1.008
15	MXEC50=−279.011+(1.026×CAEC50)+(272.921×AQ50)	0.992	6.426	1.037
30	MXEC50=−249.576+(1.053×CAEC50)+(236.791×AQ50)	0.991	6.669	1.000
45	MXEC50=−233.926+(1.063×CAEC50)+(219.256×AQ50)	0.987	8.715	1.008
15	MXEC75=−904.654+(0.982×CAEC75)+(923.454×AQ75)	0.989	23.70	1.010
30	MXEC75=−779.087+(1.050×CAEC75)+(743.868×AQ75)	0.984	28.59	1.002
45	MXEC75=−660.791+(1.087×CAEC75)+(601.488×AQ75)	0.974	37.73	1.029

^a^ 3M2B: 3-methyl-2-butanone, MX: mixture, IA: independent action, CA: concentration addition

^b^ duration of exposure in min

^c^ coefficient of determination (r^2^)

^d^ standard error of the estimate

^e^ variance inflation factor

Chemicals from several specific reaction mechanisms and each of the four MOA noted above were tested with 3M2B in this study, so the approach appears robust. With data and analyses already completed, future reports will demonstrate that this approach consistently produces high-quality MLR equations for other “chemical A” selections tested in an A-B series.

The MLR equations for the 15- and 30-min data are presented to allow modeling the dynamics of mixture toxicity over time. For example, the Lambert model [36] has a time component that will enable data for each exposure duration to be analyzed together and for response surface analysis. Dynamic mixture toxicity modeling may provide further insights into chemical mechanisms or modes of toxic action.

While MLR equations for estimating 45-min MX toxicity at the EC_10_ were also generated, they are not presented, even though r^2^ and VIF values were similar to those noted (Table 5). This choice was made because concentration selection was not designed to emphasize low-level effects. The general approach taken by Escher and colleagues [11] is amenable to generating MLR equations at low CRC effect levels.

## Conclusions

Mixture toxicity for binary combinations of 3M2B and an electrophile produced the following results: 1) the predicted IA EC_50_ was always more toxic than that for CA, 2) combined effects obtained were classified into one of seven groupings based on three relevant criteria, 3) non-sham MXs having toxicity consistent with CA were classified as being “coincident” with CA rather than fitting the mechanism-based CA definition, and 4) high-quality MLR equations for estimating mixture toxicity of 3M2B-containing binary mixtures were obtained for both IA and CA at each exposure duration and effect level. Conceptually, the approach can be used with other model organisms and in low-effects level testing.

## Supporting information

S1 Fig Concentration-response curve (CRC) plot. The plotted curves are for 3-methyl-2-butanone (3M2B) alone, hydroxypropyl methacrylate alone (HPM), the 3M2B-HPM mixture (MX) and the predicted concentration addition (CA) and independent action (IA) models. Note that the MX toxicity is consistent with that predicted for IA but more toxic than predicted for CA. Each CRC is given in 3M2B-equivalent concentrations (the upper X-axis). The lower X-axis depicts the CRC for actual HPM alone concentrations.
(TIF)

S2 Fig Concentration-response curve (CRC) plot. The plotted curves are for 3-methyl-2-butanone (3M2B) alone, 2,6-dichloro-4-nitropyridine alone (26D4NP), the 3M2B-26D4NP mixture (MX) and the predicted concentration addition (CA) and independent action (IA) models. Note that the MX curve crosses the IA curve but is more toxic than predicted for CA. Each CRC is given in 3M2B-equivalent concentrations (the upper X-axis). The lower X-axis depicts the CRC for actual 26D4NP alone concentrations.
(TIF)

S3 Fig Concentration-response curve (CRC) plot. The plotted curves are for 3-methyl-2-butanone (3M2B) alone, ethyl fluoroacetate alone (EFAC), the 3M2B-EFAC mixture (MX) and the predicted concentration addition (CA) and independent action (IA) models. Note that the MX toxicity curve crosses both the IA and CA curves. Each CRC is given in 3M2B-equivalent concentrations (the upper X-axis). The lower X-axis depicts the CRC for actual EFAC alone concentrations.
(TIF)

S4 Fig Concentration-response curve (CRC) plot. The plotted curves are for 3-methyl-2-butanone (3M2B) alone, ethyl bromoacetate alone (EBAC), the 3M2B-EBAC mixture (MX) and the predicted concentration addition (CA) and independent action (IA) models. Note that the MX curve shows toxicity at (i.e., lower portion of curve) or less than (i.e., upper portion of curve) that predicted for IA but more toxic than predicted for CA. Each CRC is given in 3M2B-equivalent concentrations (the upper X-axis). The lower X-axis depicts the CRC for actual EBAC alone concentrations.
(TIF)

S5 Fig Concentration-response curve (CRC) plot. The plotted curves are for 3-methyl-2-butanone (3M2B) alone, 3,4-hexanedione alone (34H), the 3M2B-34H mixture (MX) and the predicted concentration addition (CA) and independent action (IA) models. Note that the MX curve shows toxicity that is less than that predicted for IA but ‘coincident’ with that predicted for CA. Each CRC is given in 3M2B-equivalent concentrations (the upper X-axis). The lower X-axis depicts the CRC for actual 34H alone concentrations.
(TIF)

S6 Fig Concentration-response curve (CRC) plot. The plotted curves are for 3-methyl-2-butanone (3M2B) alone, methyl-2-chloroacetoacetate alone (M2CA), the 3M2B-M2CA mixture (MX) and the predicted concentration addition (CA) and independent action (IA) models. Note that the MX curve shows toxicity that is less than that predicted for both IA and CA. Each CRC is given in 3M2B-equivalent concentrations (the upper X-axis). The lower X-axis depicts the CRC for actual M2CA alone concentrations.
(TIF)
