# Supplementary figures and images for: Equations for estimating binary mixture toxicity: 3-methyl-2-butanone with a series of electrophiles
Source: PLoS One. 2024 Jul 3;19(7):e0306382. doi: 10.1371/journal.pone.0306382 (PMC11221661; doi:10.1371/journal.pone.0306382)

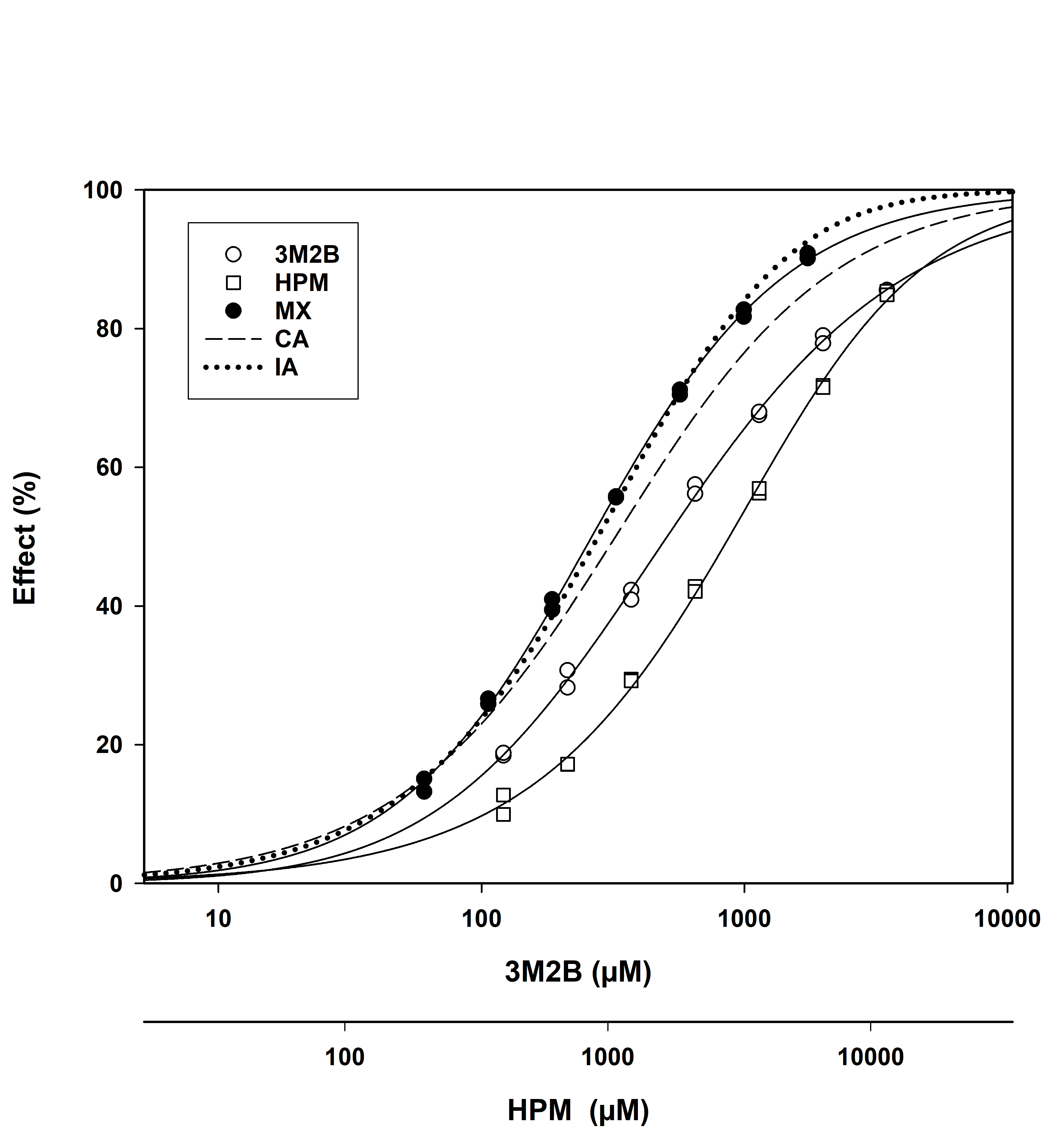

Supplement: S1 Fig — The plotted curves are for 3-methyl-2-butanone (3M2B) alone, hydroxypropyl methacrylate alone (HPM), the 3M2B-HPM mixture (MX) and the predicted concentration addition (CA) and independent action (IA) models. Note that the MX toxicity is consistent with that predicted for IA but more toxic than predicted for CA. Each CRC is given in 3M2B-equivalent concentrations (the upper X-axis). The lower X-axis depicts the CRC for actual HPM alone concentrations. (TIF) [file pone.0306382.s001.tif]

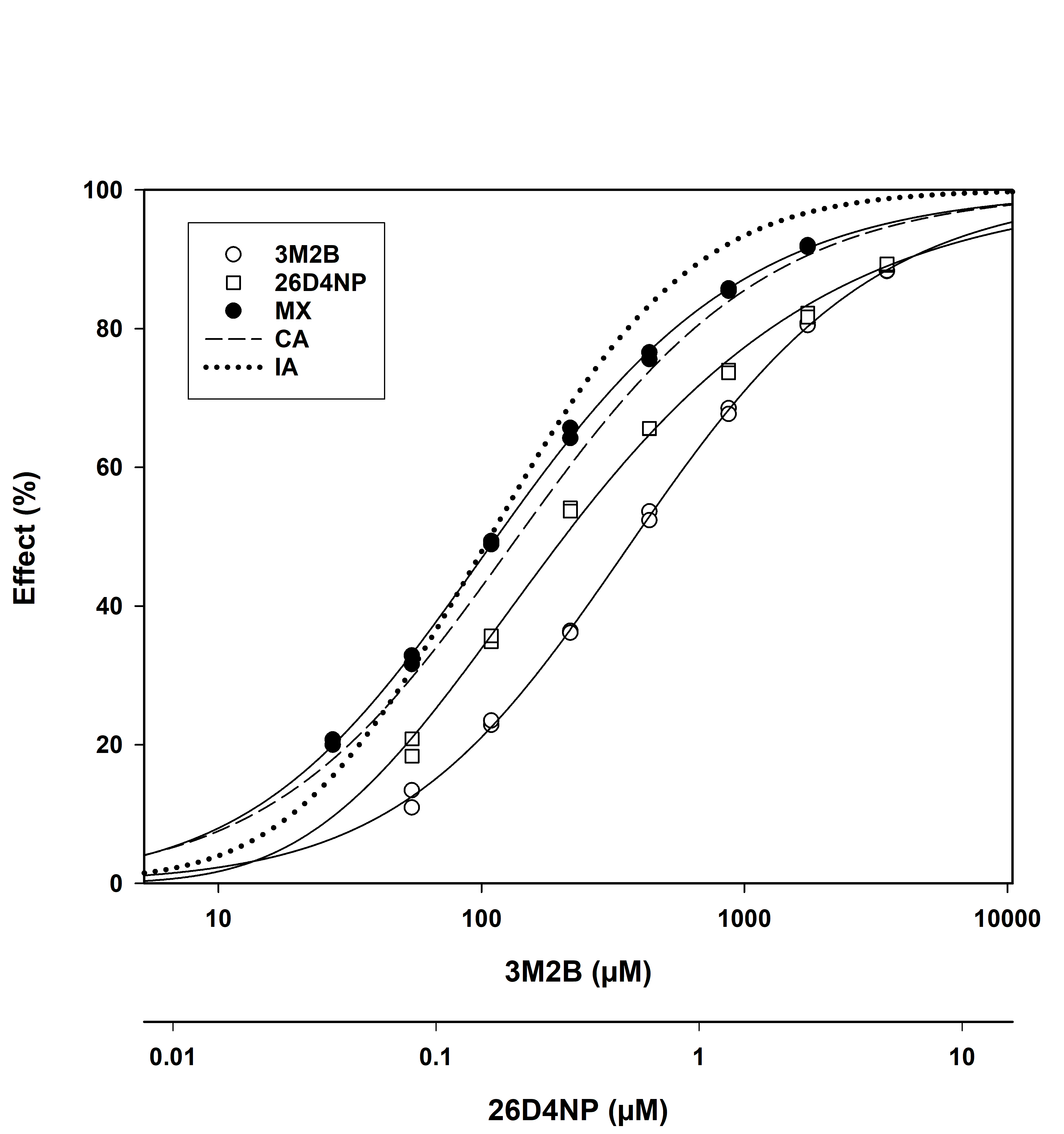

Supplement: S2 Fig — The plotted curves are for 3-methyl-2-butanone (3M2B) alone, 2,6-dichloro-4-nitropyridine alone (26D4NP), the 3M2B-26D4NP mixture (MX) and the predicted concentration addition (CA) and independent action (IA) models. Note that the MX curve crosses the IA curve but is more toxic than predicted for CA. Each CRC is given in 3M2B-equivalent concentrations (the upper X-axis). The lower X-axis depicts the CRC for actual 26D4NP alone concentrations. (TIF) [file pone.0306382.s002.tif]

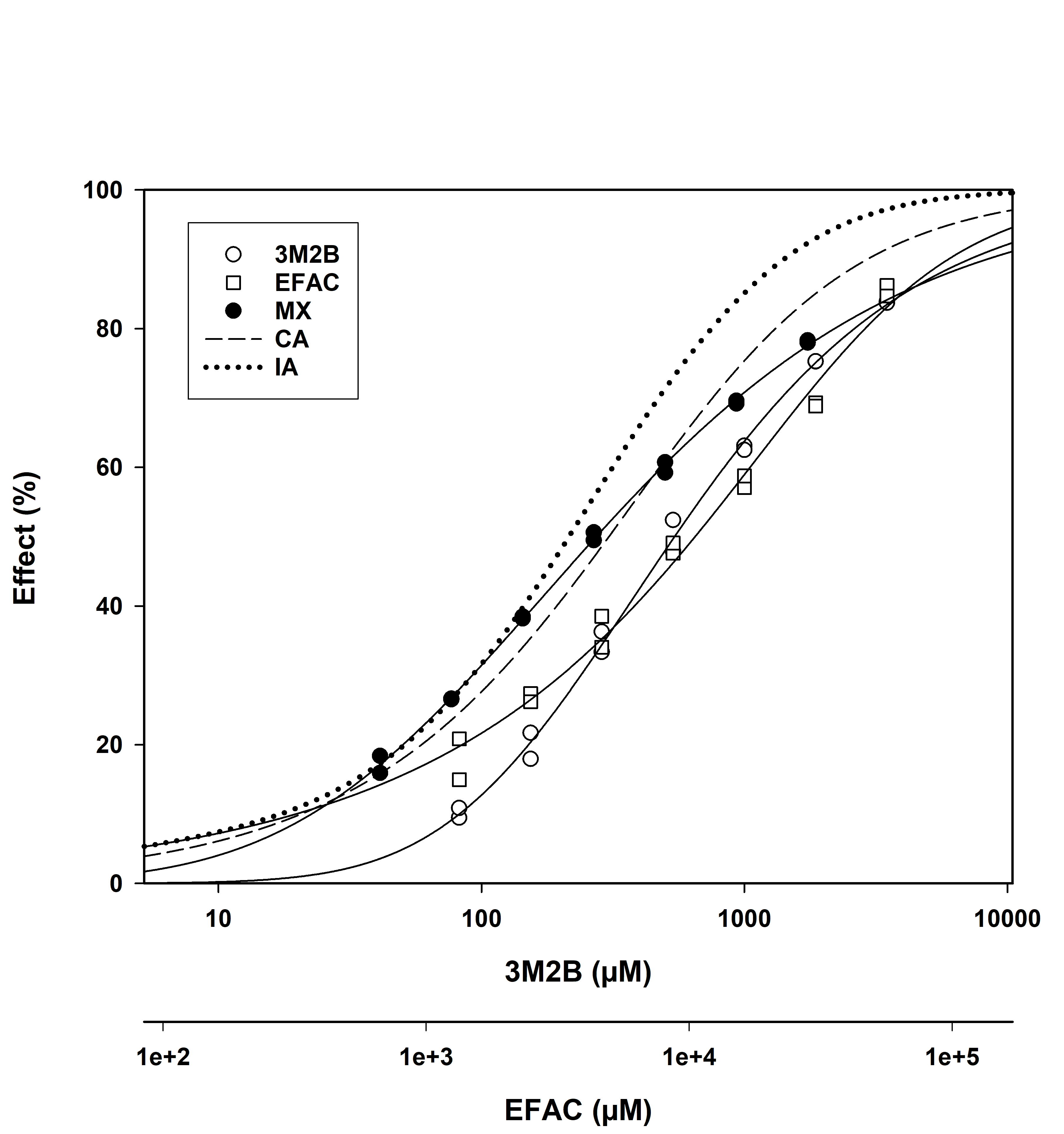

Supplement: S3 Fig — The plotted curves are for 3-methyl-2-butanone (3M2B) alone, ethyl fluoroacetate alone (EFAC), the 3M2B-EFAC mixture (MX) and the predicted concentration addition (CA) and independent action (IA) models. Note that the MX toxicity curve crosses both the IA and CA curves. Each CRC is given in 3M2B-equivalent concentrations (the upper X-axis). The lower X-axis depicts the CRC for actual EFAC alone concentrations. (TIF) [file pone.0306382.s003.tif]

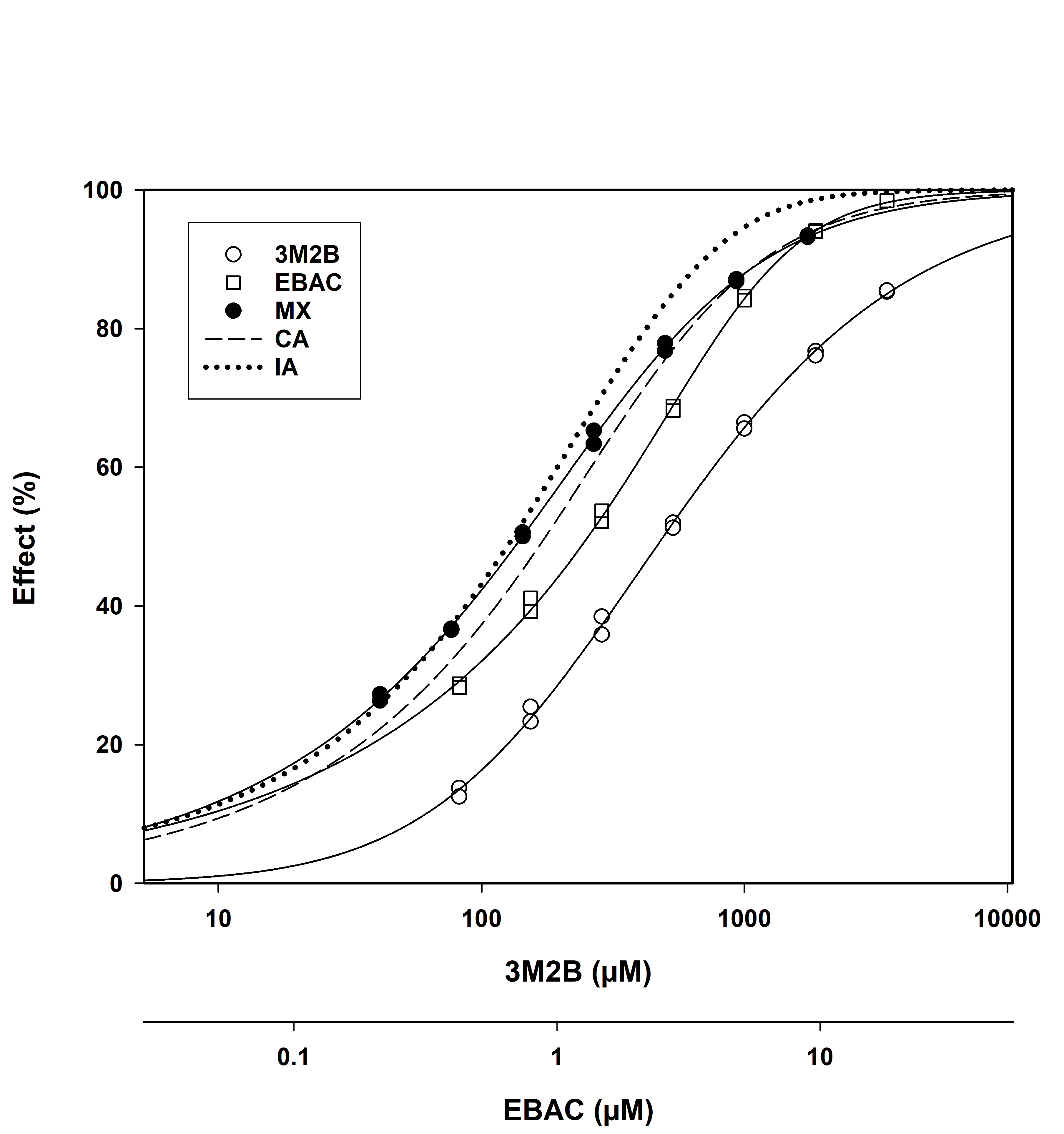

Supplement: S4 Fig — The plotted curves are for 3-methyl-2-butanone (3M2B) alone, ethyl bromoacetate alone (EBAC), the 3M2B-EBAC mixture (MX) and the predicted concentration addition (CA) and independent action (IA) models. Note that the MX curve shows toxicity at (i.e., lower portion of curve) or less than (i.e., upper portion of curve) that predicted for IA but more toxic than predicted for CA. Each CRC is given in 3M2B-equivalent concentrations (the upper X-axis). The lower X-axis depicts the CRC for actual EBAC alone concentrations. (TIF) [file pone.0306382.s004.tif]

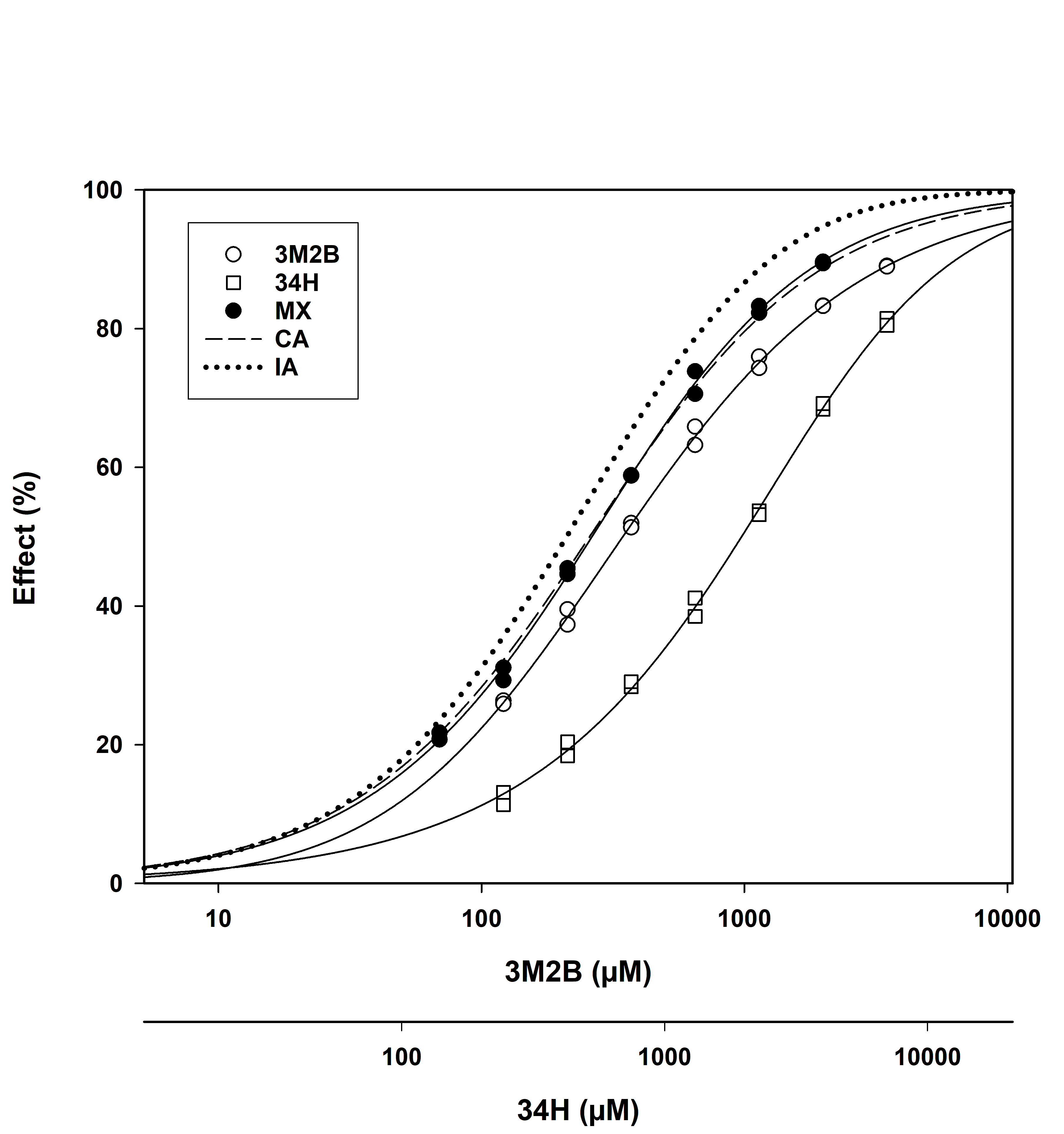

Supplement: S5 Fig — The plotted curves are for 3-methyl-2-butanone (3M2B) alone, 3,4-hexanedione alone (34H), the 3M2B-34H mixture (MX) and the predicted concentration addition (CA) and independent action (IA) models. Note that the MX curve shows toxicity that is less than that predicted for IA but ‘coincident’ with that predicted for CA. Each CRC is given in 3M2B-equivalent concentrations (the upper X-axis). The lower X-axis depicts the CRC for actual 34H alone concentrations. (TIF) [file pone.0306382.s005.tif]

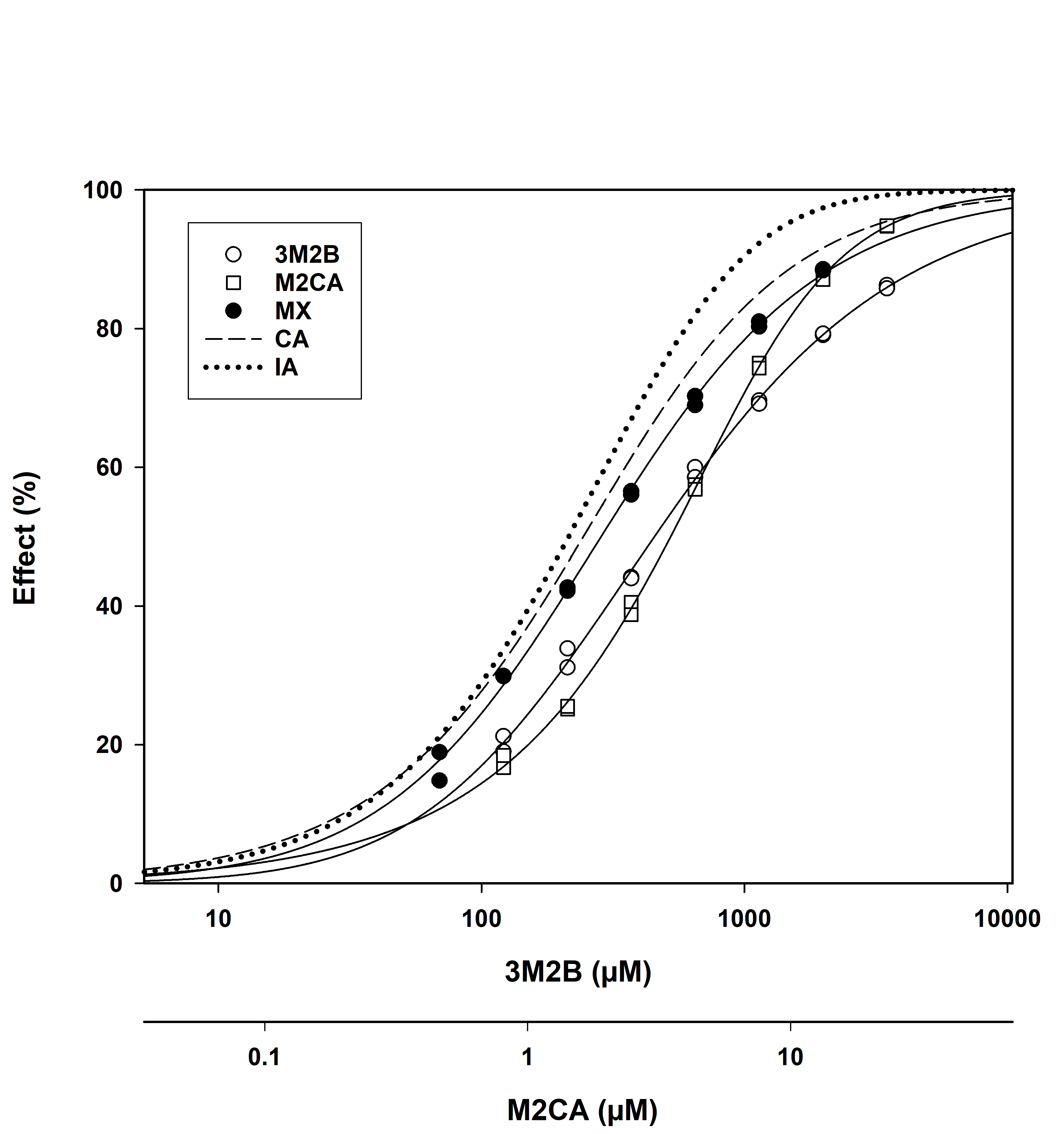

Supplement: S6 Fig — The plotted curves are for 3-methyl-2-butanone (3M2B) alone, methyl-2-chloroacetoacetate alone (M2CA), the 3M2B-M2CA mixture (MX) and the predicted concentration addition (CA) and independent action (IA) models. Note that the MX curve shows toxicity that is less than that predicted for both IA and CA. Each CRC is given in 3M2B-equivalent concentrations (the upper X-axis). The lower X-axis depicts the CRC for actual M2CA alone concentrations. (TIF) [file pone.0306382.s006.tif]
